# Human Papillomavirus Positivity and Cognitive Function in Older U.S. Adults: A Cross-Sectional Population-Based Study

**DOI:** 10.3390/pathogens14050508

**Published:** 2025-05-21

**Authors:** Thomas J. Farrer, Jonathan D. Moore, Brinley N. Zabriskie, Morgan Chase, Chris H. Miller, Shawn D. Gale, Dawson W. Hedges

**Affiliations:** 1 Idaho WWAMI Medical Education Program, University of Idaho, 875 Perimeter Dr., Moscow, ID 83844, USA; tfarrer@uidaho.edu; 2 Department of Data Analytics and Information Systems, Utah State University, Logan, UT 84322, USA; brinley.zabriskie@usu.edu; 3 The Neuroscience Center, Brigham Young University, Provo, UT 84602, USA; mchase11@student.byu.edu (M.C.); shawn_gale@byu.edu (S.D.G.); dawson_hedges@byu.edu (D.W.H.); 4 Department of Psychology, California State University, Fresno, CA 93740, USA; chmiller@csufresno.edu; 5 Department of Psychology, Brigham Young University, Provo, UT 84602, USA

**Keywords:** human papillomavirus (HPV), cognitive function, older adults, NHANES, survey-weighted regression, cross-sectional design

## Abstract

Prior research has reported an association between human papillomavirus (HPV) seropositivity and dementia or Alzheimer’s disease. This study aimed to cross-sectionally investigate the association between HPV seropositivity and cognitive function in older adults. The data used for this study were from the 2011–2014 National Health and Nutrition Examination Survey (NHANES) cycles. To account for the complex survey design and missing data, we applied survey-weighted regression models to each imputed dataset, using multiple imputation techniques. Across all cognitive test outcomes, no statistically significant association was observed between HPV status and cognitive performance after controlling for covariates. These findings suggest that there may not be a significant association between HPV infection and cognitive scores in this NHANES sample. Stakeholders, including policymakers and healthcare providers, should consider these findings in their professional decision-making. Future research should investigate the association between HPV seropositivity and cognition using other samples in order to further characterize the association between HPV and cognitive function in older adults.

## 1. Introduction

Viruses have been associated with an increased risk of neurodegenerative disease [1] and cognitive decline [2,3]. A systemic inflammatory response associated with infection is a putative mechanism of action underlying associations between viral infection and cognitive function and neurodegenerative disease. Among other mechanisms, the inflammatory response may have deleterious effects to the blood–brain barrier and result in increased CNS penetrance by pathogens [3]. Further, the inflammatory response may interfere with cellular function and increase pathological biomarkers of Alzheimer’s disease and other diseases [3]. Additionally, this inflammatory response could be linked with oxidative stress, possibly resulting in the signs and symptoms of Alzheimer’s disease [4]. Extensive research has focused on a wide variety of infectious agents and their impact on the brain [5].

A deoxyribonucleic acid virus with over 200 different genotypes [6], human papillomavirus (HPV) infects approximately 31 percent of men globally [7] and 12 percent of women globally, but with high regional variation [8]. Approximately 70 percent of low- and middle-income countries do not have national HPV vaccination programs, resulting in higher prevalence of HPV among these populations [9]. Despite the lack of HPV vaccine programs in many regions, many healthcare professionals in several countries advocate for HPV vaccines, but there is a need to increase education and information available to both healthcare professionals and patients in order to improve HPV vaccine coverage [10].

HPV can infect the anogenital tract [6] and has been found in brain tissue [6]. HPV is also the primary cause of cervical cancer and can cause other forms of cancer as well as cutaneous and genital warts [6]. Although many HPV infections do not result in cancer or genital warts, a 2018 study estimated a prevalence of 2.23% of genital warts in US men aged 18 to 59 years [11]. Accumulating evidence suggests that HPV may be associated with cardiovascular disease (CVD), including CVD-related mortality [12,13]. While the mechanism underlying this association is still unclear, long-term systemic inflammation from the HPV infection may be related to atherosclerosis, which may explain why in some studies, though not others, HPV seropositivity has been linked to stroke [12,13,14].

In addition to these associations, HPV subtype 71 has been associated with Alzheimer’s disease (odds ratio [OR] = 3.56, adjusted *p* = 0.02) in a study investigating viral genetic material in human DNA using data from the Alzheimer’s Disease Sequencing Project [15]. HPV subtype 71 is generally thought to be a low-risk HPV subtype for cancer [16,17], although some data suggest that there may be an association with more malignant cancers in some populations [18]. HPV 71 has a low prevalence, with estimates from Mexico [18], Thailand [19,20], and the United States [21] all being less than or approximately 1% of the population of women in each respective country. Additionally, in their review, Talwar and colleagues identified an interaction between multiple viruses, including HPV, and eight genes potentially associated with Alzheimer’s disease [4]. HPV primarily interacted with the epidermal growth factor receptor [4], a factor receptor thought to be involved in Alzheimer’s disease through its association with memory impairment in invertebrate models, correlations with amyloid-beta plaque aggregation, and potential neurotoxicity and neuroinflammation [22].

Caused by HPV subtypes 6 and 11, genital warts have been associated with an increased risk of dementia. For example, a study using Taiwan’s National Health Insurance Research Database with a 15-year follow-up found that a positive diagnosis of genital warts was associated with risk for both vascular dementia and Alzheimer’s disease [23]. In this study, genital warts in male patients conferred a higher risk of dementia than in female patients, although genital warts were associated with a higher risk of dementia in both women and men [23]. A study utilizing national biobanks to investigate the risk for neurodegenerative diseases associated with exposure to viruses suggested that since genital warts are caused by HPV, it may be possible to reduce neurodegeneration risk through vaccination [1].

Because of HPV’s high worldwide prevalence [7,8], its potential role in dementia, and dementia’s listing as a World Health Organization global health priority [24], our primary objective was to evaluate the cross-sectional association between HPV positivity in saliva regardless of HPV subtype and cognitive function determined by performance on four tests of cognitive function in a sample of older adults aged 60 years and older from the United States, while adjusting for several possibly confounding covariates, including as a secondary objective marker of systemic inflammation that could affect the relationship between HPV and cognitive function. We hypothesized that there would be an association between HPV positivity and cognitive function in our sample. To our knowledge, this is the first cross-sectional study to evaluate the association between HPV positivity regardless of the presence of genital warts or HPV subtype and cognitive function in older adults.

## 2. Materials and Methods

### 2.1. Design Overview and Sample

The National Health and Nutrition Examination Survey (NHANES) is a national sample collected by the National Center for Health Statistics within the United States Centers for Disease Control and Prevention [25], and our study utilized a cross-sectional design based on these data. Information collected for NHANES includes demographic data and health and nutrition statuses collected during in-person interviews and physical examinations. The NHANES uses a complex sampling methodology, including multistage probability sampling, to collect data from approximately 5000 people annually, which is then used to estimate the entire non-institutionalized United States population and disseminates in 2-year cycles [25]. Data for all variables were taken from the 2011–2014 NHANES cycles because these cycles were the most recent cycles containing both information about HPV status and cognitive function. There were 27,763 total individuals selected for inclusion for NHANES from 2011 to 2014, with 19,931 who completed the survey and 19,151 who were examined. Age ranged from 14 to 80 years. We restricted the data to participants aged 60 years or older with non-zero survey weights, resulting in 3472 observations with an age range from 60 to 80 years. NHANES obtains National Center for Health Statistics Ethics Review Board (ERB) approval for each of their cycles of data [26]. This study was reported following the Strengthening the Reporting of Observational Studies in Epidemiology (STROBE) guidelines [27].

### 2.2. Exposure of Interest

The exposure of interest for this study was oral HPV. Presence of HPV in an oral rinse provided an estimate of the prevalence of oral HPV in the United States population. The oral HPV result was categorized as positive versus negative.

### 2.3. Outcomes of Interest

The outcomes of interest were performance on the Word List Memory subtest from the Consortium to Establish a Registry for Alzheimer’s Disease (CERAD) battery (CERAD) [28], the Animal Fluency Test (AFT) [29], and the Digit Symbol Substitution test (DSST) [30]. Descriptions of these tests can be found on the CDC’s website [31] and in the paper describing the CERAD battery [28].

Briefly, the CERAD Word Learning task is a measure of verbal learning and memory [28]. This test involves reading aloud a list of 10 unrelated words and then recalling as many as possible over three different trials. Rather than analyzing performance on each individual trial, we evaluated total acquisition over the three trials, with a maximum score of 30, which is considered a measure of learning. After the three trials and completion of other cognitive tasks, participants were asked to recall as many words as they could from the learning trials. Just as learning words (e.g., acquisition) from the word list is sensitive to cognitive impairment, the ability to recall information after a delay has been shown to be sensitive to cognitive impairment and dementia [32,33]. The delayed recall trial has a maximum score of 10. The delayed recall portion of the test was conducted after the other two tests (AFT and DSST) were completed [34].

The AFT is a measure of executive functioning and verbal fluency during which participants are asked to generate as many names of animals as possible in one minute. A participant’s score is counted as one point for every unique animal generated [29]. Some have described this task as an “access” aspect of executive function since it is associated with efficient access to information stored in long-term memory [35]; because of the nature of the task, it has also been described as an executive measure of “generativity” [36].

The DSST evaluates working memory, mental processing speed, and sustained attention. This test involves presenting participants with a document that has a key of nine numbers that are matched with various symbols. Participants are given two minutes to copy as many of the corresponding symbols as possible for each of the 133 boxes next to the numbers. A participant’s score is the number of correct matches, with 133 being the highest possible score.

To summarize, four cognitive functioning measures were used as dependent variables: CERAD Learning, CERAD Delayed Recall, the AFT, and the DSST. On each of these measures, a higher score represents better performance.

### 2.4. Covariates

To control against possible confounding, we preselected several covariates based on their potential associations with both the exposure and outcome variables, with levels of categorical variables having been determined by NHANES. We included age, sex, smoking status (never, former, current), body mass index (BMI; <18.5 kg/m^2^, 18.5–24.9 kg/m^2^, 25.0–29.9 kg/m^2^, ≥30 kg/m^2^), and mean systolic blood pressure (mmHg), as well as history of coronary heart disease (“Has a doctor or other health professional ever told you that you had coronary heart disease?”; yes/no), and stroke (“Has a doctor or other health professional ever told you that you had a stroke?”; yes/no). In addition, we included the following demographic variables: sex (female, male), race/Hispanic origin (Mexican American, Other Hispanic, Non-Hispanic White, Non-Hispanic Black, Other), the socioeconomic factors of educational attainment (less than high-school graduate, high-school graduate, some college or above), and poverty index ratio (above poverty, near poverty, and below poverty). Poverty index ratio (PIR) was measured by dividing family income by poverty guidelines (a continuous measure provided by NHANES) that we recategorized based on previous research (above poverty: PIR ≥ 2.0, near poverty: 1.0–1.9, and below poverty: <1.0) [37]. We also included several inflammatory markers: the systemic immune inflammation index (SII) [(platelets × neutrophils)/lymphocytes], the neutrophil-to-lymphocyte ratio (NLR), the lymphocyte-to-monocyte ratio (LMR), and the platelet-to-lymphocyte ratio (PLR) [38].

### 2.5. Statistical Analysis

The final sample size was 3472 participants, and all analyses were conducted using the R Statistical Software (Version 4.4.2) [39]. All reported confidence intervals are based on a 95% confidence level.

#### 2.5.1. Imputation Method

We performed multiple imputation using the jomo R package, which implements joint modeling multiple imputation under a multivariate normal model fitted via Markov Chain Monte Carlo (MCMC) sampling [40,41]. This approach was selected for its flexibility in handling complex data structures, including multilevel models and datasets containing both continuous and categorical variables. Unlike single imputation or ad hoc methods such as complete case analysis—which can lead to biased estimates and loss of efficiency—multiple imputation accounts for uncertainty due to missing data by creating multiple plausible datasets, analyzing each one separately, and combining the results using Rubin’s rules [41,42]. Consistent with NHANES guidance, records with missing values were not dropped, as doing so can lead to incorrect variance estimation under the complex survey design [43]. The imputation model assumes that data are missing at random (MAR)—that is, the probability of missingness depends on observed variables but not on the missing values themselves.

Imputation was conducted on the full dataset (N = 19,151), prior to subsetting by age. This was necessary because the survey package requires access to the full sampling design—including strata, clusters, and sampling weights—before subsetting in order to compute design-consistent variance estimates [43]. Subsetting beforehand would risk distorting the survey design structure and result in incorrect standard error estimation.

Prior to finalizing the imputation model, we considered several auxiliary variables that could plausibly help predict HPV status [44]. However, each of these candidate variables had over 94% missing data, which made them unsuitable for inclusion, as their sparse data made them unlikely to improve prediction and could have negatively affected model performance.

We also conducted a diagnostic run to evaluate MCMC convergence properties before generating imputations. Trace plots and posterior density plots indicated poor convergence for coefficients associated with the four inflammatory markers initially included in the model. These convergence issues appeared to result from high multicollinearity among the markers. To improve model stability, we retained only one inflammatory marker—neutrophil-to-lymphocyte ratio (NLR)—in the final imputation model. NLR was chosen based on its empirical support in the literature linking it to dementia outcomes [45,46]. After this adjustment, convergence diagnostics improved substantially. Following a burn-in of 10,000 iterations, we generated 10 multiply imputed datasets, each separated by 1000 iterations, and used these in all subsequent analyses.

#### 2.5.2. Survey Design and Regression Modeling

To account for both the complex survey design and multiple imputation, we fit survey-weighted regression models across the imputed datasets and combined the results using Rubin’s rules [41]. For each of the 10 imputed datasets, a survey design object was created using the full NHANES sample (N = 19,151) with appropriate clustering, stratification, and sampling weights, using the svydesign function from the survey package. Each design object was then subset to include only participants aged 60 and older. We fit separate survey-weighted regression models for each of the four cognitive outcomes using the svyglm function from the survey package, incorporating the set of predictors described above.

We initially aimed to fit a multivariate linear regression model, as the four cognitive test scores used as outcomes are moderately to highly correlated, and joint modeling could account for shared variance and improve efficiency. However, current statistical software—including the survey package recommended by NHANES—does not support multivariate outcome models that incorporate survey weights, clustering, and stratification. To maintain valid variance estimation under the complex sampling structure, we followed NHANES guidance and instead fit separate survey-weighted regression models for each cognitive outcome.

Regression coefficients and variance estimates were stored for each imputed dataset and pooled using standard multiple imputation combining rules, drawing code from the MIcombine function in the mitools package and using Rubin’s rules [41,47]. To evaluate the statistical significance of interaction terms and other nested model comparisons, we tested whether the addition of parameters significantly improved model fit using a multivariate Wald-type F test appropriate for multiply imputed data. Specifically, we compared nested models across all imputed datasets by fitting each model separately using svyglm with full survey design adjustments and then pooling the results using a D1 statistic [48]. This approach accounts for both within- and between-imputation variability and returns an overall F statistic, numerator and denominator degrees of freedom, and a pooled *p*-value. The procedure was implemented in custom code adapted from the mitml and mitools R packages and applied to each cognitive outcome separately [47,49]. This allowed us to test whether including interaction terms or additional predictors significantly improved model fit under the complex survey design and missing data assumptions.

## 3. Results

### 3.1. Summary Statistics

Data for 3472 people were used in the analysis. Weighted summary statistics, including means, standard deviations, Pearson correlation coefficients, and proportions, are presented in Table 1 for all participants that had data recorded for the variable of interest (prior to imputation). These summary statistics were calculated with the survey R package using survey-weighted estimates to account for the complex sampling design, which involved stratification, clustering, and unequal probabilities of selection [50]. Survey weights adjust for oversampling of certain groups and non-response, ensuring that the results are representative of the broader population rather than just the sample. Unweighted statistics may misrepresent the population distribution, leading to biased results. We also report the proportion of missing values for each variable.

After accounting for the study design, the prevalence of oral HPV positivity was estimated to be 7.9%. The mean age was 69.6 years (SD = 6.8). Women account for 54.9% of the target population, and non-Hispanic Whites for 77.4%. The percentage who had completed some college or above was 59%, those who had never smoked was 49.9%, and those who had a BMI classification of obese was 37%. Mean systolic blood pressure was 132 mm Hg (SD = 19.3), while 7.7% had previously been told they had a stroke.

Of the 3472 people, 39% (1361 people) had data recorded for every variable. HPV had the most missing data with 53.1% missing, as shown in Table 1. The outcome variables had between 9.8% and 13.2% missing data, followed by the NLR inflammatory variable with 4.8% missing. The remaining covariates had less than 4% missing data. Among the measures of cognitive function, CERAD Learning and CERAD Delayed Recall exhibited the strongest correlation (r = 0.75), followed by DSST and AFT (r = 0.53). Lastly, to address right-skewness, the NLR inflammatory predictor was log-transformed prior to the imputation and analysis.

### 3.2. Imputation Model Results

For the multiple imputation model, visual inspection of trace and posterior density plots from the convergence diagnostic run (Figures S1 and S2) supported adequate mixing and stationarity of the MCMC chains. After reducing the set of inflammatory markers to include only NLR, convergence diagnostics improved substantially, confirming the stability of the final imputation model that was then used to create 10 imputed datasetss.

### 3.3. Survey Regression Model Results

**Figure 1:**
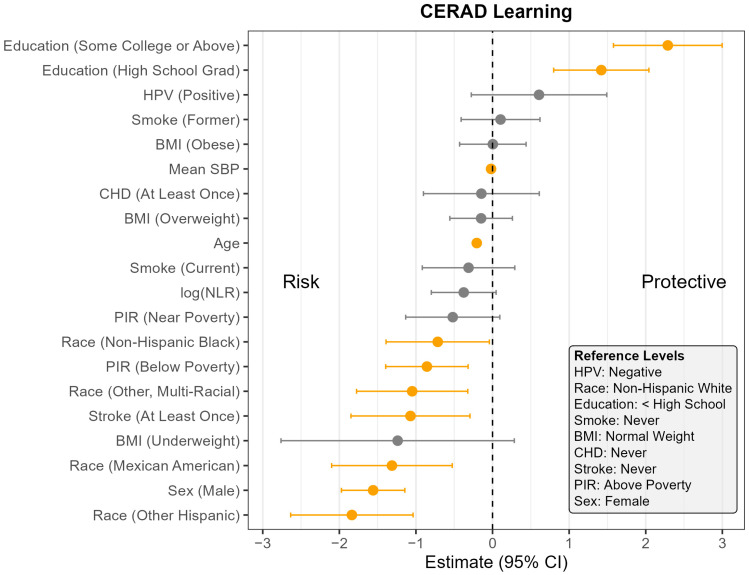
Coefficient estimates and 95% confidence intervals from the survey-weighted regression model predicting CERAD Learning scores among U.S. adults aged 60 and older, NHANES 2011–2014. Estimates reflect the adjusted association between each predictor and CERAD Learning score, based on multiply imputed data and accounting for the complex sampling design. Predictors with confidence intervals that do not include zero are highlighted in orange. Reference levels for categorical variables are indicated in the lower corner of the plot. CHD—Coronary Heart Disease, HPV—Human Papillomavirus, BMI—Body Mass Index, SBP—Systolic Blood Pressure, log NLR—Neutrophil-to-Lymphocyte Ratio, PIR—Poverty Income Ratio, NHANES—National Health and Nutrition Examination Survey, CERAD—Consortium to Establish a Registry for Alzheimer’s Disease, U.S.—United States. The vertical dotted line indicates the cut off between positive and negative effect.

**Figure 2:**
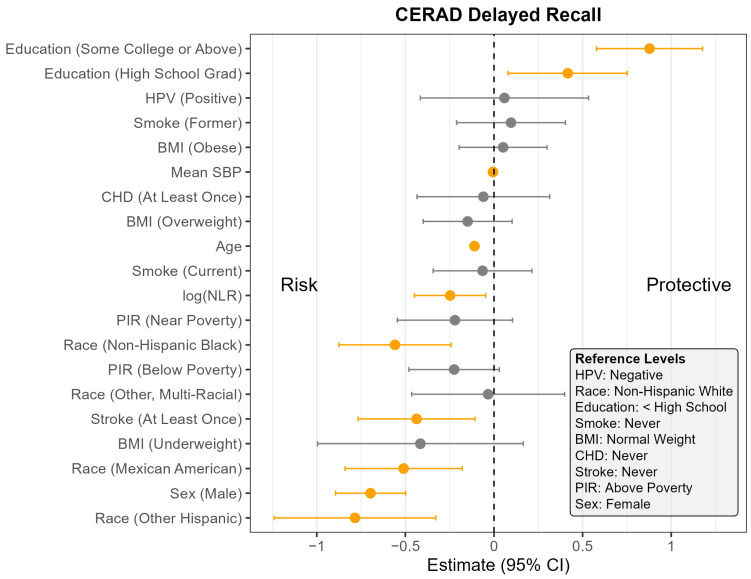
Coefficient estimates and 95% confidence intervals from the survey-weighted regression model predicting CERAD Delayed Recall scores among U.S. adults aged 60 and older, NHANES 2011–2014. Estimates reflect the adjusted association between each predictor and CERAD Delayed Recall score, based on multiply imputed data and accounting for the complex sampling design. Predictors with confidence intervals that do not include zero are highlighted in orange. Reference levels for categorical variables are indicated in the lower corner of the plot. CHD—Coronary Heart Disease, HPV—Human Papillomavirus, BMI—Body Mass Index, SBP—Systolic Blood Pressure, log NLR—Neutrophil-to-Lymphocyte Ratio, PIR—Poverty Income Ratio, NHANES—National Health and Nutrition Examination Survey, CERAD—Consortium to Establish a Registry for Alzheimer’s Disease, U.S.—United States. The vertical dotted line indicates the cut off between positive and negative effect.

**Figure 3:**
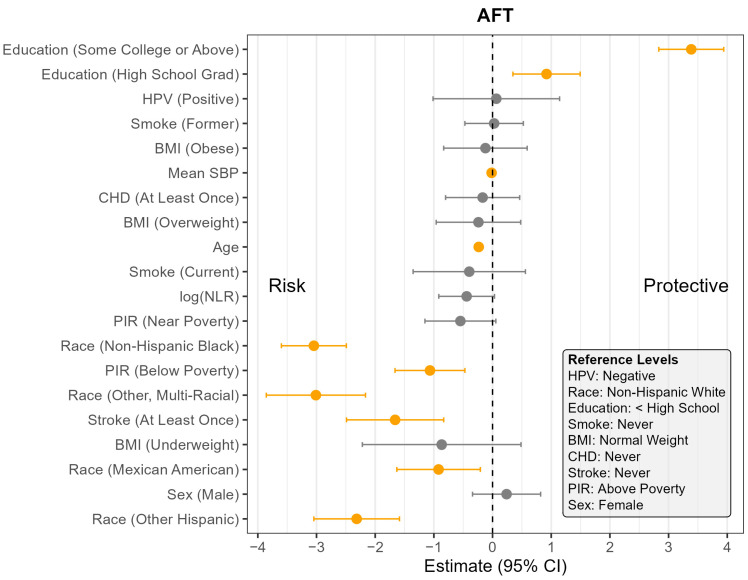
Coefficient estimates and 95% confidence intervals from the survey-weighted regression model predicting AFT scores among U.S. adults aged 60 and older, NHANES 2011–2014. Estimates reflect the adjusted association between each predictor and AFT score, based on multiply imputed data and accounting for the complex sampling design. Predictors with confidence intervals that do not include zero are highlighted in orange. Reference levels for categorical variables are indicated in the lower corner of the plot. CHD—Coronary Heart Disease, HPV—Human Papillomavirus, BMI—Body Mass Index, SBP—Systolic Blood Pressure, log NLR—Neutrophil-to-Lymphocyte Ratio, PIR—Poverty Income Ratio, NHANES—National Health and Nutrition Examination Survey, AFT—Animal Fluency Test, U.S.—United States. The vertical dotted line indicates the cut off between positive and negative effect.

**Figure 4:**
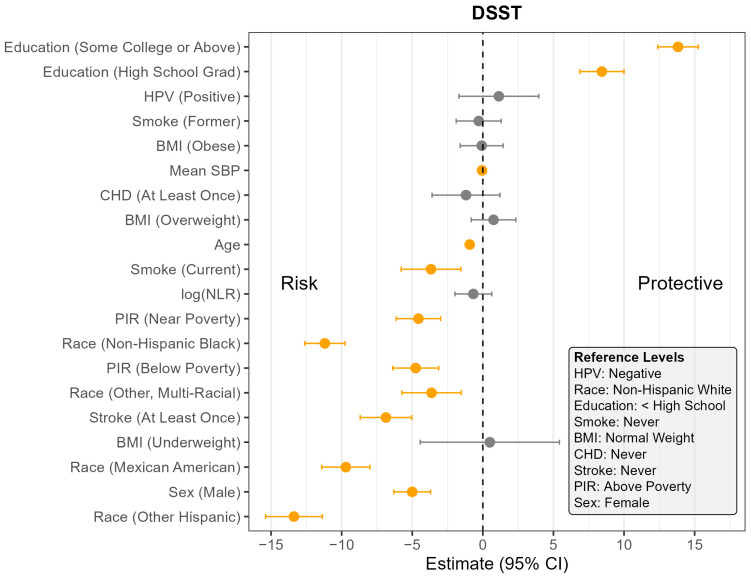
Coefficient estimates and 95% confidence intervals from the survey-weighted regression model predicting DSST among U.S. adults aged 60 and older, NHANES 2011–2014. Estimates reflect the adjusted association between each predictor and DSST, based on multiply imputed data and accounting for the complex sampling design. Predictors with confidence intervals that do not include zero are highlighted in orange. Reference levels for categorical variables are indicated in the lower corner of the plot. CHD—Coronary Heart Disease, HPV—Human Papillomavirus, BMI—Body Mass Index, SBP—Systolic Blood Pressure, log NLR—Neutrophil-to-Lymphocyte Ratio, PIR—Poverty Income Ratio, NHANES—National Health and Nutrition Examination Survey, DSST—Digit Symbol Substitution Test, U.S.—United States. The vertical dotted line indicates the cut off between positive and negative effect.

The pooled F-test evaluating the interaction between NLR and HPV status yielded relatively large *p*-values across all cognitive outcomes (CERAD Learning: *p* = 0.07; CERAD Delayed Recall: *p* = 0.07; AFT: *p* = 0.44; DSST: *p* = 0.65), suggesting the association between NLR and cognitive outcomes did not meaningfully differ by HPV status. Consequently, the remaining analyses were conducted using models without the interaction term. Supplementary Table S1 presents the full regression results for all four models, and Figure 1, Figure 2, Figure 3 and Figure 4 display the corresponding coefficient plots in the main text.

Across all four outcomes, there was no statistically significant association between HPV status and cognitive performance after adjusting for other covariates (Figure 1, Figure 2, Figure 3 and Figure 4). Similarly, NLR was not significantly associated with cognitive outcomes in most models, with one exception: CERAD Delayed Recall. To put this in context, a 25% increase in NLR (e.g., from the median of 2.1 to 2.6) would be associated with a 0.01-to-0.10-point decrease in average score in the CERAD Delayed Recall (Figure 2). While a 25% increase in NLR would not be clinically significant on the CERAD Delayed Recall, this association warrants additional investigation. Other covariates that did not show significant associations across the models are BMI and CHD.

In contrast, several factors demonstrated consistent and significant negative associations with cognitive performance across all four outcomes (Figure 1, Figure 2, Figure 3 and Figure 4). Individuals who did not complete high school performed significantly worse on average than those with higher levels of education. Those identifying as Mexican American, Other Hispanic, or Non-Hispanic Black had lower average performance compared to Non-Hispanic White participants. Individuals who reported a history of stroke also performed worse on average than those without a stroke diagnosis. Cognitive scores declined with increasing age, and higher mean SBP was associated with lower cognitive performance.

Gender and poverty status were also associated with differences in cognitive outcomes. Men scored significantly lower than women on CERAD Learning, CERAD Delayed Recall, and DSST, but no significant gender difference was observed for AFT. Individuals with a PIR below the poverty threshold performed worse on CERAD Learning, AFT, and DSST than those above the poverty threshold, although there was no significant difference for CERAD Delayed Recall. Those identifying as Other or Multi-Racial had significantly lower scores on CERAD Learning, AFT, and DSST, but not on CERAD Delayed Recall, compared to Non-Hispanic White individuals. In addition, current smoking was associated with significantly worse performance on DSST compared to those who had never smoked. A PIR classified as near poverty was also associated with lower DSST scores compared to those above the poverty threshold.

## 4. Discussion

In this community-based sample from a high-income country, adjusted main-effect models showed no association between HPV seropositivity and CERAD Learning, CERAD Delayed Recall, AFT, and DSST in adults aged 60 years or older. These results broadly differ from those of Lin et al., who found a significant association between genital warts, which is caused by HPV types 6 and 11, and incident dementia in adults over aged 50 years [23]. In interpreting these differences, several factors require consideration. Lin et al. used incident dementia as the outcome variable [23], whereas we used cognitive function in older adults. In addition, Lin et al. used genital warts as the exposure variable [23], whereas we used HPV positivity in salivary samples regardless of the presence of genital warts, which could reflect a difference in exposure intensity. Further, we assessed the cross-sectional association between HPV seropositivity and cognitive function but not longitudinal dementia incidence, as in Lin et al. [23], who found no difference in dementia incidence between patients positive and negative for genital warts until after three years. Further, our sample differed in age from the Lin et al. sample (mean age 69.6 years versus 42 years, respectively) [23]. Similarly, our results differ from Tejeda et al., who found an association between HPV type 71 and Alzheimer’s disease [15]. Tejeda et al. included just one serotype of HPV, whereas we investigated associations between HPV regardless of subtype and cognitive function [15]. Further, Tejeda et al. used Alzheimer’s disease as an outcome [15] in contrast to our use of performance on a battery of cognitive tasks.

Our findings showing no associations between HPV and cognitive function in older adults should in no way discourage the use of HPV vaccines. Some of the primary reasons for low HPV vaccine uptake include a disparity in HPV vaccine availability, a lack of understanding of HPV and the HPV vaccine, and health infrastructure issues [10]. By systematically addressing these issues through methods such as better training, government policies, and increased accessibility [10], the prevalence of HPV and cervical cancer can decrease with improvement in health across the world.

Although not the primary focus of our study, we explored the role of peripheral inflammatory markers in cognitive function by including them as covariates in our models. Due to high correlations among markers and issues with model convergence during imputation, we excluded three of the four markers—SII, LMR, and PLR—from the final analyses. Among the markers examined, only NLR was retained. While the association between NLR and cognitive outcomes did not meaningfully differ by HPV status, NLR was significantly associated with performance on the CERAD Delayed Recall task, but not with CERAD Learning, AFT, or DSST. In this regard, Tondo et al. [51] found that in patients with a mean age of 76.5 years with amnestic mild cognitive impairment, the NLR predicted a diagnosis of dementia over a follow-up from two to five years [51]. The possible association between a marker of peripheral inflammation and memory function in older adults warrants additional research, as it may also be indicative of an association between immune function, cognitive function, and risk for dementia [52]. Further, additional research into associations between biomarkers of systemic inflammation and cognitive function and dementia require more research, particularly as some markers of systemic inflammation have been associated with depression [53]. Several associations in our adjusted models provide insight into the range of factors that may be involved in cognitive function in adults aged 60 years and older. While we did not find associations between HPV and cognitive function, we did find associations between gender and performance on the CERAD Learning, CERAD Delayed, and DSST measures, such that women did better than men on these measures, a finding consistent with other studies [54,55]. Current smoking, comparatively low educational attainment, and PIR near poverty were also associated with lower cognitive function.

### 4.1. Limitations

Several factors require consideration when interpreting these findings. The cross-sectional nature of the study implies that there was no control over the timing of infection or re-infection and no control over symptom severity. Thus, generalizing to the broader population should be undertaken with caution, as we were unable to restrict sample inclusion based on these factors. Future research should try to take timing of infection and symptom severity into consideration as factors in their sample or as variables in their statistical models.

An additional limitation of this study is the substantial amount of missing data for HPV status, which was one of our primary variables of interest. Over half of the HPV data points were missing, and although we used multiple imputation to handle this missingness, the results should still be interpreted with caution. This missing information potentially weakens the study’s ability to detect associations involving HPV status and may impact the robustness of our findings related to this variable. Future research with more complete HPV data could offer clearer insights into its role and help determine whether the associations we observed remain consistent when missing data are minimized.

Another limitation of this study is the use of four separate models for each of the four response variables. These response variables are interrelated and analyzing them independently does not fully capture the potential dependencies among them. For example, the correlation coefficient for CERAD Learning and CERAD Delayed Recall was 0.75. Ideally, a multivariate model would have been more appropriate, as it would allow for joint modeling of all four outcomes, taking into account their correlations. However, due to the complex survey design of the data we used, with features such as stratification, clustering, and weighting, currently available software does not readily support multivariate modeling that accounts for these complex design elements. While separate models provide valuable insights, they may lead to inefficiencies or biased estimates, particularly when the response variables are correlated. Future advancements in statistical software or methods for complex survey data would enhance the robustness of analyses by allowing for multivariate modeling that accommodates the intricate design of survey data.

We also note that this study was based on data from a high-income country. It is possible that there may be associations between HPV status and cognitive function in other regions that differ in socioeconomic conditions, medical variables, and exposure to other infectious diseases, highlighting the need for additional work in other geographical regions investigating associations between cognitive function and infectious diseases among dementia in older adults.

### 4.2. Conclusions

The primary objective of this study was to investigate the association between HPV positivity and cognitive test scores using the NHANES data. However, we did not find any significant association between HPV status and cognitive function, as assessed by the CERAD Learning, CERAD Delayed, AFT, and DSST cognitive tests in this sample of older adults from a high-income nation. Stakeholders, including policymakers and healthcare providers, could consider these findings in their professional decision-making. Additional research is needed to evaluate the role of HPV positivity, including at varying exposure levels, in cognitive function and dementia in older adults. Future research should also investigate the association between HPV seropositivity and cognition using other samples to compare findings.

In addition, the inflammatory marker NLR was significantly associated with performance on the CERAD Delayed Recall task, but not with CERAD Learning, AFT, or DSST. Although NLR was only associated with one aspect of memory and no other cognitive measures, it is interesting. Prior work has suggested that one mechanism by which viruses may affect cognitive function is through an ongoing inflammatory response. We believe this should be a topic of future research not only to determine if measures such as NLR may relate to the underlying mechanism of infections contributing to the risk of dementia, but also whether they act as predictors that could possibly be a target of prevention.

## Figures and Tables

**Table 1:** Weighted summary statistics for all participants that had data recorded for the variable of interest, NHANES 2011–2014 (*n* = 3472).

	NHANES Source.	Variable		Weighted Estimate	Weighted SD	Percent Missing
	ORXHPV	HPV Seropositivity				53.1
			Negative	92.1	–	
			Positive	7.9	–	
Outcome Variables	CFDDS	DSST		51.8	16.9	13.2
	CFDAST	AFT		17.9	5.8	10.4
	CFDCSR	CERAD Delayed Recall		6.1	2.4	10.0
	CFDCST1 + CFDCST2 + CFDCST3	CERAD Learning		19.5	4.7	9.8
	LBDNENO/LBDLYMNO	NLR		2.5	1.6	4.8
	(BPXSY1 + BPXSY2 + BPXSY3 + BPXSY4)/4	Mean SBP		132.0	19.3	3.9
	BMXBMI	BMI				2.2
			Obese	37.0	–	
			Overweight	36.1	–	
			Normal Weight	25.3	–	
			Underweight	1.6	–	
	INDFMPIR	PIR				2.1
			Above Poverty	68.1	–	
			Near Poverty	16.7	–	
			Below Poverty	15.2	–	
	MCQ160C	CHD				0.8
			Never	90.2	–	
			At Least Once	9.8	–	
	MCQ160F	Stroke				0.2
			Never	92.3	–	
			At Least Once	7.7	–	
	DMDEDUC2	Education				0.2
			Some College or Above	59.0	–	
			High School Grad	22.2	–	
			<High School	18.8	–	
	If SMQ020 = “No”, then “Never” If SMQ020 = “Yes” & SMQ040 = “No”, then “Former” If SMQ020 = “Yes” & SMQ040 = “Yes”, then “Current”	Smoke				0.1
			Never	49.9	–	
			Former	38.8	–	
			Current	11.3	–	
	RIDAGEYR	Age		69.6	6.8	0.0
	RIDRETH1	Race				0.0
			Non-Hispanic White	77.4	–	
			Non-Hispanic Black	9.1	–	
			Other Race or Multi-Racial	5.9	–	
			Mexican American	3.8	–	
			Other Hispanic	3.9	–	
	RIAGENDR	Gender				0.0
			Female	54.9	–	
			Male	45.1	–	

Grey shaded lines distinguish outcome variables used in this study. The weighted estimate reflects means for continuous variables and percentages for categorical variables. Standard deviations are presented only for continuous variables. AFT—Animal Fluency Test, BMI—Body Mass Index, BPXSY1–BPXSY4—Systolic Blood Pressure readings 1–4, CERAD—Consortium to Establish a Registry for Alzheimer’s Disease, CHD—Coronary Heart Disease, CFDAST—Animal Fluency Test, CFDCSR—CERAD Delayed Recall, CFDCST1–3—CERAD Immediate Learning Trials 1–3, CFDDS—Digit Symbol Substitution Test, DSST—Digit Symbol Substitution Test, HPV—Human Papillomavirus, INDFMPIR—Income-to-Poverty Ratio (Poverty Income Ratio), LBDLYMNO—Lymphocyte Count, LBDNENO—Neutrophil Count, MCQ160C—Ever told you had coronary heart disease, MCQ160F—Ever told you had a stroke, NLR—Neutrophil-to-Lymphocyte Ratio, NHANES—National Health and Nutrition Examination Survey, ORXHPV—Laboratory Variable for HPV Seropositivity, PIR—Poverty Income Ratio, RIDAGEYR—Age in Years, RIDRETH1—Race/Ethnicity, RIAGENDR—Sex, SBP—Systolic Blood Pressure, SMQ020—Ever Smoked 100 Cigarettes in Life, and SMQ040—Currently Smoke.

## Data Availability

The original data presented in the study are openly available on the NHANES website at https://wwwn.cdc.gov/nchs/nhanes/continuousnhanes/. Data were accessed on 12 June 2024.

## Supplementary Materials

The following supporting information can be downloaded at: https://www.mdpi.com/article/10.3390/pathogens14050508/s1, Figure S1: Trace plots of MCMC samples for the 18 regression coefficients from the convergence diagnostic run for the imputation model. These plots show the sampled values of regression coefficients across 10,000 MCMC iterations generated by the jomo.MCMCchain R function, which was used to evaluate convergence of the imputation model. The visual inspection of trace plots indicates stable mixing and no apparent trends or drifts over time, supporting the adequacy of the burn-in and total iteration settings used in the final imputation procedure.; Figure S2: Density plots of MCMC samples for the 18 regression coefficients from the convergence diagnostic run for the imputation model. These plots display the marginal distributions of sampled regression coefficients obtained from 10,000 iterations of the convergence-checking model (from the jomo.MCMCchain R function). The unimodal and stable densities suggest that the MCMC algorithm mixes well and supports convergence of the joint imputation model. These diagnostics informed the choice of burn-in and iteration parameters for the final imputation procedure. Table S1: Survey-weighted linear regression results for four cognitive outcomes among U.S. adults aged 60 and older, NHANES 2011–2014. Estimates reflect the average change in cognitive score associated with each predictor, adjusting for all other variables in the model. Results are based on multiply imputed data and account for the complex NHANES sampling design.
