# Supplementary material for: Human Papillomavirus Positivity and Cognitive Function in Older U.S. Adults: A Cross-Sectional Population-Based Study
Source: Pathogens. 2025 May 21;14(5):508. doi: 10.3390/pathogens14050508 (PMC12114561; doi:10.3390/pathogens14050508)
Supplement: Supplementary file 1 [file pathogens-14-00508-s001.zip › pathogens-3637968-supplementary.pdf]

Supplementary Material

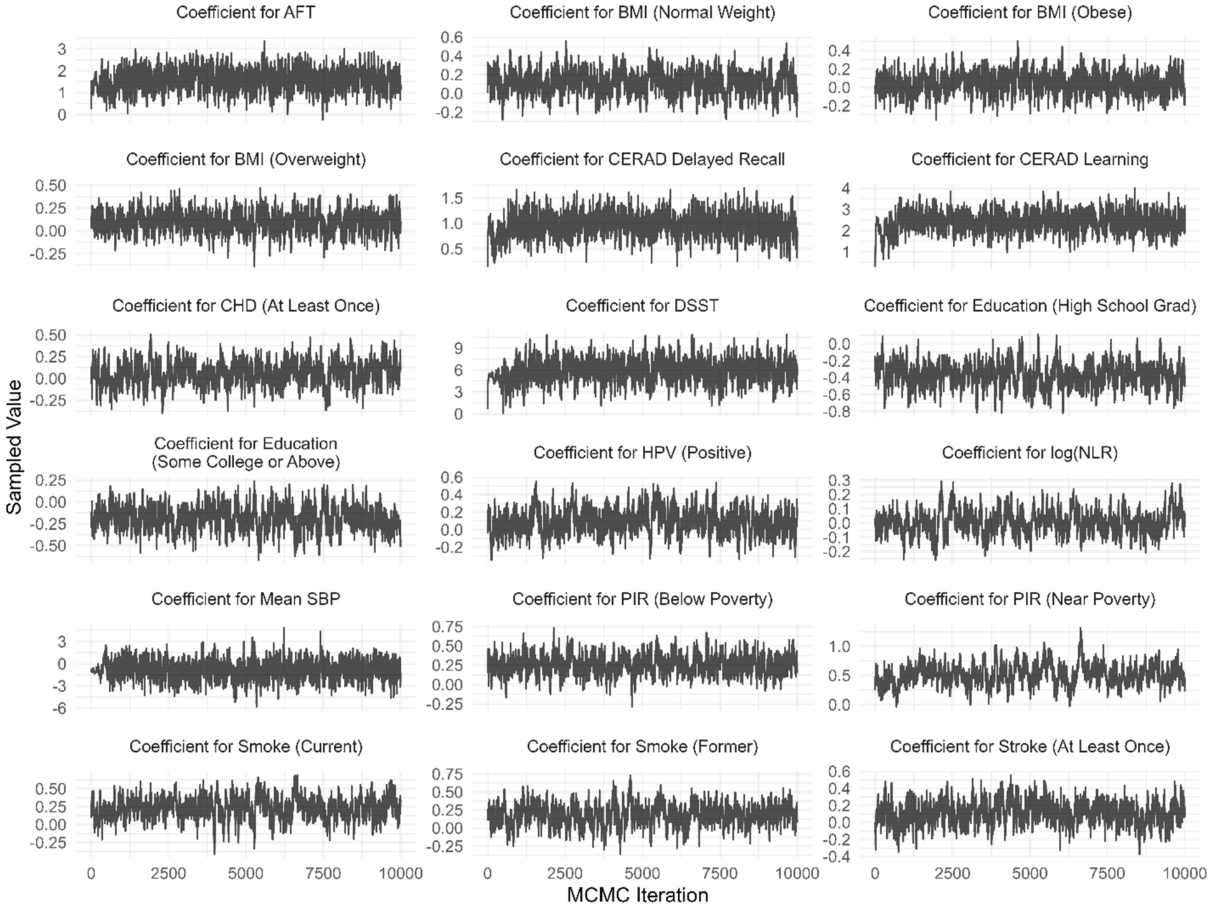

Figure S1. Trace plots of MCMC samples for the 18 regression coefficients from the convergence diagnostic run for the imputation model. These plots show the sampled values of regression coefficients across 10,000 MCMC iterations generated by the jomo.MCMCchain R function, which was used to evaluate convergence of the imputation model. The visual inspection of trace plots indicates stable mixing and no apparent trends or drifts over time, supporting the adequacy of the burn-in and total iteration settings used in the final imputation procedure.

AFT – Animal Fluency Test, BMI – Body Mass Index, CHD – Coronary Heart Disease, CERAD – Consortium to Establish a Registry for Alzheimer's Disease, DSST – Digit Symbol Substitution Test, HPV – Human Papillomavirus, log NLR – Neutrophil-to-Lymphocyte Ratio, PIR – Poverty-to-Income Ratio, SBP – Systolic Blood Pressure, MCMC – Markov Chain Monte Carlo

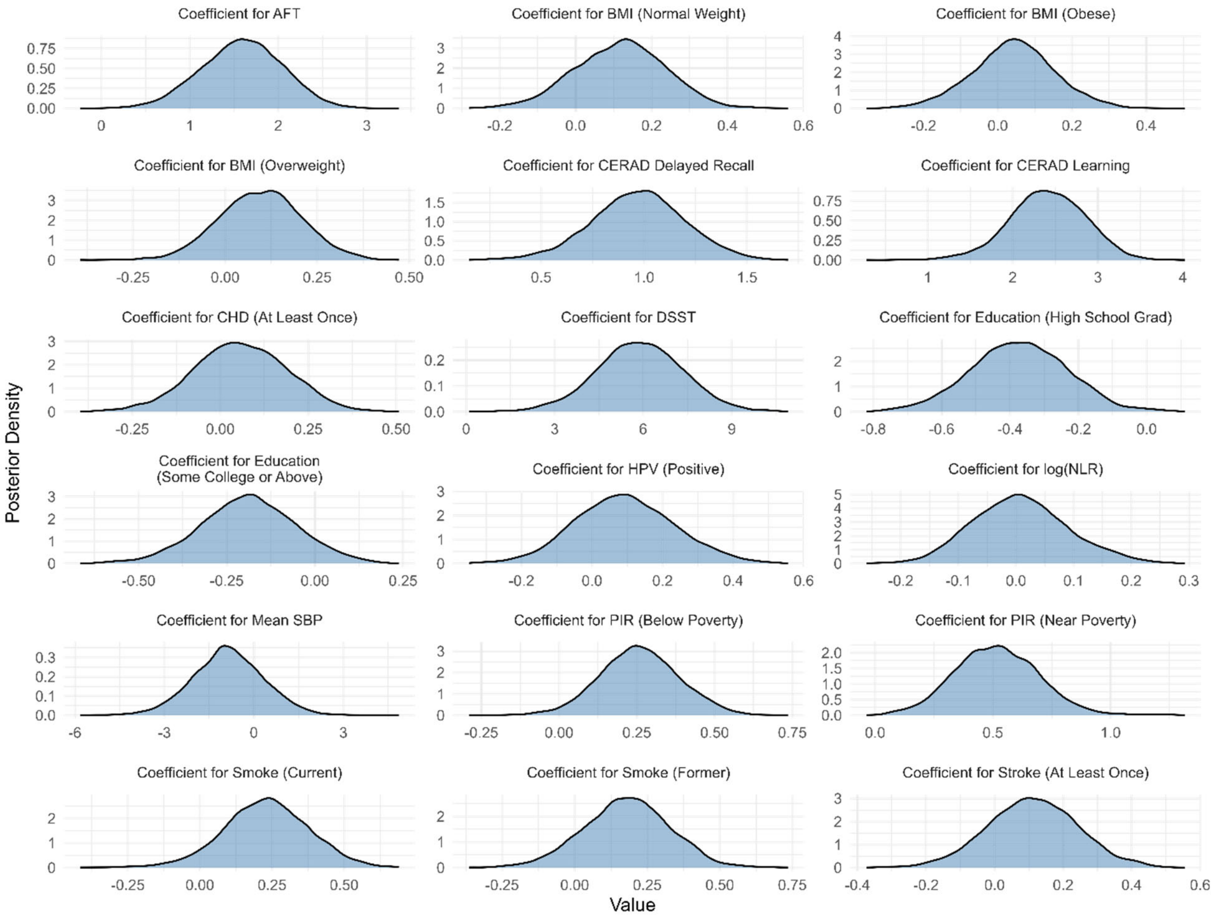

Figure S2. Density plots of MCMC samples for the 18 regression coefficients from the convergence diagnostic run for the imputation model. These plots display the marginal distributions of sampled regression coefficients obtained from 10,000 iterations of the convergence-checking model (from the jomo.MCMCchain R function). The unimodal and stable densities suggest that the MCMC algorithm mixes well and supports convergence of the joint imputation model. These diagnostics informed the choice of burn-in and iteration parameters for the final imputation procedure.

AFT – Animal Fluency Test, BMI – Body Mass Index, CHD – Coronary Heart Disease, CERAD – Consortium to Establish a Registry for Alzheimer's Disease, DSST – Digit Symbol Substitution Test, HPV – Human Papillomavirus, log NLR – Neutrophil-to-Lymphocyte Ratio, PIR – Poverty-to-Income Ratio, SBP – Systolic Blood Pressure, MCMC – Markov Chain Monte Carlo

Table S1. Survey-weighted linear regression results for four cognitive outcomes among U.S. adults aged 60 and older, NHANES 2011-2014. Estimates reflect the average change in cognitive score associated with each predictor, adjusting for all other variables in the model. Results are based on multiply imputed data and account for the complex NHANES sampling design.

	Estimate (95% CI)			
**Variable**	**CERAD Learning**	**CERAD Delayed Recall**	**AFT**	**DSST**
**Intercept**	33.62 (30.34, 36.91)	13.66 (12.19, 15.12)	33.60 (30.65, 36.54)	111.08 (102.47, 119.70)
**HPV (Reference Level: Negative)**				
Positive	0.61 (-0.28, 1.49)	0.06 (-0.42, 0.53)	0.06 (-1.01, 1.14)	1.13 (-1.70, 3.96)
**Age**	-0.21 (-0.24, -0.17)	-0.11 (-0.13, -0.09)	-0.24 (-0.28, -0.19)	-0.93 (-1.03, -0.83)
**Race (Reference Level: Non-Hispanic White)**				
Other Hispanic	-1.84 (-2.64, -1.04)	-0.78 (-1.24, -0.33)	-2.32 (-3.05, -1.59)	-13.38 (-15.39, -11.37)
Mexican American	-1.31 (-2.10, -0.53)	-0.51 (-0.84, -0.18)	-0.92 (-1.63, -0.21)	-9.70 (-11.41, -8.00)
Non-Hispanic Black	-0.72 (-1.39, -0.04)	-0.56 (-0.88, -0.24)	-3.04 (-3.60, -2.49)	-11.19 (-12.61, -9.77)
Other, Multi-Racial	-1.05 (-1.78, -0.32)	-0.03 (-0.46, 0.40)	-3.01 (-3.86, -2.17)	-3.63 (-5.73, -1.53)
**Education (Reference Level: < High School)**				
High School Grad	1.42 (0.80, 2.04)	0.42 (0.08, 0.75)	0.92 (0.35, 1.49)	8.43 (6.86, 10.00)
Some College or Above	2.29 (1.58, 3.00)	0.88 (0.58, 1.18)	3.39 (2.83, 3.94)	13.82 (12.39, 15.26)
**Smoke (Reference Level: Never)**				
Former	0.10 (-0.41, 0.62)	0.10 (-0.21, 0.40)	0.03 (-0.47, 0.53)	-0.29 (-1.89, 1.30)
Current	-0.31 (-0.92, 0.29)	-0.06 (-0.34, 0.21)	-0.40 (-1.35, 0.56)	-3.67 (-5.79, -1.56)
**BMI (Reference Level: Normal Weight)**				
Underweight	-1.24 (-2.76, 0.28)	-0.42 (-1.00, 0.16)	-0.87 (-2.22, 0.49)	0.49 (-4.43, 5.42)
Overweight	-0.15 (-0.56, 0.26)	-0.15 (-0.40, 0.10)	-0.24 (-0.96, 0.48)	0.76 (-0.82, 2.34)
Obese	0.00 (-0.43, 0.44)	0.05 (-0.20, 0.30)	-0.12 (-0.83, 0.59)	-0.08 (-1.60, 1.44)
**CHD (Reference Level: Never)**				
At Least Once	-0.15 (-0.90, 0.61)	-0.06 (-0.43, 0.31)	-0.17 (-0.80, 0.46)	-1.19 (-3.60, 1.22)
**Stroke (Reference Level: Never)**				
At Least Once	-1.07 (-1.85, -0.29)	-0.44 (-0.77, -0.11)	-1.66 (-2.49, -0.83)	-6.86 (-8.69, -5.04)
**Mean SBP**	-0.02 (-0.03, -0.01)	-0.01 (-0.01, -0.00)	-0.02 (-0.03, -0.01)	-0.06 (-0.09, -0.02)
**PIR (Reference Level: Above Poverty)**				

	Near Poverty	-0.52 (-1.13, 0.10)	-0.22 (-0.55, 0.10)	-0.55 (-1.15, 0.06)	-4.56 (-6.13, -2.99)
	Below Poverty	-0.86 (-1.39, -0.32)	-0.22 (-0.48, 0.03)	-1.07 (-1.66, -0.47)	-4.76 (-6.39, -3.13)
**Log(NLR)**		-0.38 (-0.80, 0.05)	-0.25 (-0.45, -0.05)	-0.44 (-0.92, 0.03)	-0.67 (-1.97, 0.63)
**Gender (Reference Level: Female)**					
	Male	-1.56 (-1.97, -1.14)	-0.70 (-0.89, -0.50)	0.24 (-0.34, 0.82)	-5.00 (-6.30, -3.70)
